# Neurobiology and Therapeutic Potential of Cyclooxygenase-2 (COX-2) Inhibitors for Inflammation in Neuropsychiatric Disorders

**DOI:** 10.3389/fpsyt.2019.00605

**Published:** 2019-09-04

**Authors:** Rickinder Sethi, Nieves Gómez-Coronado, Adam J. Walker, Oliver D’Arcy Robertson, Bruno Agustini, Michael Berk, Seetal Dodd

**Affiliations:** ^1^Department of Psychiatry, Western University, London, ON, Canada; ^2^Unidad de Gestión Clinica Salud Mental, Hospital Universitario Virgen del Rocio, Sevilla, Spain; ^3^IMPACT Strategic Research Centre, Deakin University, Geelong, VIC, Australia; ^4^University Hospital Geelong, Barwon Health, Geelong, VIC, Australia; ^5^Department of Psychiatry, The University of Melbourne, Parkville, VIC, Australia; ^6^Orygen, The National Centre of Excellence in Youth Mental Health, Parkville, VIC, Australia; ^7^Department of Psychiatry, Florey Institute of Neuroscience and Mental Health, Parkville, VIC, Australia

**Keywords:** depression, bipolar disorder, schizophrenia, obsessive compulsive disorder, autism spectrum disorder, psychiatry, inflammation, cyclooxygenase-2 inhibitors

## Abstract

Neuropsychiatric disorders, such as depression, bipolar disorder, schizophrenia, obsessive-compulsive disorder, and neurodevelopmental disorders such as autism spectrum disorder, are associated with significant illness burden. Accumulating evidence supports an association between these disorders and inflammation. Consequently, anti-inflammatory agents, such as the cyclooxygenase-2 inhibitors, represent a novel avenue to prevent and treat neuropsychiatric illness. In this paper, we first review the role of inflammation in psychiatric pathophysiology including inflammatory cytokines’ influence on neurotransmitters, the hypothalamic–pituitary–adrenal axis, and microglial mechanisms. We then discuss how cyclooxygenase-2-inhibitors influence these pathways with potential therapeutic benefit, with a focus on celecoxib, due to its superior safety profile. A search was conducted in PubMed, Embase, and PsychINFO databases, in addition to Clinicaltrials.gov and the Stanley Medical Research Institute trial registries. The results were presented as a narrative review. Currently available outcomes for randomized controlled trials up to November 2017 are also discussed. The evidence reviewed here suggests cyclooxygenase-2 inhibitors, and in particular celecoxib, may indeed assist in treating the symptoms of neuropsychiatric disorders; however, further studies are required to assess appropriate illness stage-related indication.

## Introduction

The immune system involves a complex array of cells, tissues, and organs working in concert to protect the body from foreign molecules at both the intracellular and extracellular level (1). Pro-inflammatory and anti-inflammatory cytokines, along with other mechanisms, balance the inflammatory response (1). External causes of inflammation include microbial or viral infections, cigarette smoking, poor dietary composition, air pollution, and trauma (both physical and psychological), among others (2). Internal causes may include ischemic events or malignancy (1). In instances where inflammatory mediators are unable to inhibit the pro-inflammatory immune reaction, a chronic inflammatory state may ensue. Chronic activation of this system can lower the allostatic load threshold, contributing to the development of neuropsychiatric disorders (2).

While monoaminergic dysregulation remains a prevailing hypothesis regarding neuropsychiatric disorders, refractory illnesses remain a significant challenge in addition to a relative paucity of novel treatment options (3). In recent years, the inflammatory model has been revisited due to the fragmented efficacy of the current management approaches. As a result, more attention is being paid to pharmacotherapies that lay outside the traditional vault of psychotropic agents such as antidepressants and antipsychotics. Cyclooxygenase-2 inhibitors, best known for their role in acute pain management, are a potent example of this pharmacological appropriation. Celecoxib and rofecoxib—selective cyclooxygenase-2 inhibitors—have been investigated for their efficacy as both stand-alone therapies and augmentation agents in psychiatry.

The purpose of this paper is twofold: first, to review the relationship between inflammation and neuropsychiatric illnesses, and second, to provide a review of randomized control trials (RCTs) that investigate the use of cyclooxygenase-2 inhibitors for the treatment of select neuropsychiatric illnesses.

## Methods

A Boolean search was conducted for literature published up to November 19, 2017. We searched PubMed, Embase, and PsychINFO databases, and the Clinicaltrials.gov and The Stanley Medical Research Institute trial registries. Search terms included are attached in Appendix A. Articles were selected for human, randomized clinical trials and treatment efficacy. The search was augmented by manually searching the references of key papers and related literature. We adhered to PRISMA guidelines and flowsheet attached in Appendix A. **Table 1** contains a summary chart of the results. The results were presented as a narrative review format.

**Table 1:** Clinical trials investigating celecoxib in neuropsychiatric disorders.

Study	Sample	Study Design	Intervention and Dosage	Outcome Measures	Findings
Muller et al. (4)	Depression, *n* = 40	Double-blind, randomized, placebo-controlled add-on trial 6 weeks	Celecoxib 400 mg + Reboxetine VS Placebo + Reboxetine	HAM-D	Favor of adjuvant celecoxib
Akhondzadeh et al. (5)	Depression, *n* = 40	Double-blind, randomized, placebo-controlled add-on trial 6 weeks	Celecoxib 400 mg + fluoxetine VS Placebo + fluoxetine	HRDS	Favor of adjuvant celecoxib
Fields et al. (6)	Depression, *n* = 2,528 Elderly cohort	Double-blind, Multicenter, randomized, placebo-controlled add-on trial 6 weeks	Celecoxib 400 mg VS Naproxen 440 mg VS Placebo	GDS, Modified Mini-Mental State Exam (3MS-E)	Not in favor of celecoxib monotherapy
Abbasi et al. (7)	Depression, *n* = 40	Double-blind, randomized, placebo-controlled add-on trial 6 weeks	Celecoxib 400 mg + sertraline VS Placebo + sertraline	HAM-D	Favor of adjuvant celecoxib
Majd et al. (8)	Depression, *n* = 30	Double-blind, randomized, placebo-controlled add-on trial 8 weeks	Celecoxib 200 mg + sertraline VS Placebo + sertraline	HAM-D, HAM-A	Not in favor of celecoxib
Jafari et al. (9)	Depression, *n* = 40 Comorbid brucellosis	Double-blind, randomized, placebo-controlled add-on trial 8 weeks	Celecoxib 200 mg + antibiotics VS Placebo + antibiotics	HDRS	Favor of adjuvant celecoxib
Mohammad et al. (10)	Depression, *n* = 52 Comorbid breast cancer	Double-blind, randomized, placebo-controlled add-on trial 6 weeks	Celecoxib 400 mg VS diclofenac 100 mg	HDRS	Favor of celecoxib monotherapy
Alamdarsaravi et al. (11)	Depression, *n* = 40 Comorbid colorectal cancer	Double-blind, randomized, placebo-controlled add-on trial 6 weeks	Celecoxib 400 mg VS Placebo	HDRS	Favor of celecoxib monotherapy
Nery et al. (12)	Bipolar disorder–depression or mixed episode, *n* = 28	Double-blind, randomized, placebo-controlled add-on trial, 6 weeks	Celecoxib 400 mg + TAU VS Placebo + TAU	HDRS	Favor of adjuvant celecoxib
Kargar et al. (13)	Bipolar disorder–mania, *n* = 35	Double-blind, randomized, placebo-controlled add-on trial,	Celecoxib 400 mg + ECT VS Placebo + ECT	YMRS, BDNF serum levels	Not in favor of celecoxib
Arabzadeh et al. (14)	Bipolar disorder – mania, *n* = 46	Double-blind, randomized, placebo-controlled add-on trial, 6 weeks	Celecoxib 400 mg + Sodium Valproate VS Placebo + Sodium Valproate	YMRS, HDRS	Favor of adjuvant celecoxib
Shalbafan et al. (15)	OCD, *n* = 54	Double-blind, randomized, placebo-controlled add-on trial 10 weeks	Celecoxib 400 mg + Fluvoxamine 200 mg VS Placebo + Fluvoxamine 200 mg	Y-BOCS	Favor of adjuvant celecoxib
Sayyah et al. (16)	OCD, *n* = 56	Double-blind, randomized, placebo-controlled add-on trial 8 weeks	Celecoxib 400 mg + Fluoxetine VS Placebo + Fluoxetine	Y-BOCS	Favor of adjuvant celecoxib
Muller et al. (17)	Schizophrenia, *n* = 50	Double-blind, randomized, placebo-controlled add-on trial 5 weeks	Celecoxib 400 mg + Risperidone VS Placebo + Risperidone	PANSS, Simpson-Angus Rating Scale of EPS (SAS)	Favor of adjuvant celecoxib
Rapaport et al. (18)	Schizophrenia, *n* = 38	Double-blind, randomized, placebo-controlled add-on trial 9 weeks	Celecoxib 400 mg + TAU VS Placebo + TAU	PANSS, Scale for the Assessment of Negative Symptoms (SANS), CGI-S, Clinical Global Impression: Improvement (CGI-I), HAM-A	Not in favor of celecoxib
Akhondzadeh et al. (19)	Schizophrenia, *n* = 60	Double-blind, randomized, placebo-controlled add-on trial 8 weeks	Celecoxib 400 mg + Risperidone VS Placebo + Risperidone	PANSS, ESRS	Favor of adjuvant celecoxib
Muller et al. (20)	Schizophrenia, *n* = 49	Double-blind, randomized, placebo-controlled add-on trial 6 weeks	Celecoxib 400 mg+ Amisulpride 200–1,000 mg VS Placebo + Amisulpride 200–1,000 mg	PANSS, CGI	Favor of adjuvant celecoxib
Muller Trial ID: 01T-418 (yet to publish)	Schizophrenia, *n* = 40	Double-blind, randomized, placebo-controlled add-on trial 8 weeks	Celecoxib 400 mg + Risperidone VS Placebo + Risperidone	PANSS, SANS, CBI, ESRS, Barnes Akathisia QOL	Not in favor of celecoxib
Zhang Trial ID: 03T-459 (yet to publish)	Schizophrenia, *n* = 250	Double-blind, randomized, placebo-controlled add-on trial 12 weeks	Celecoxib 400 mg + Risperidone VS Placebo + Risperidone	PANSS, BPRS, SANS, ICG, WCST, N-back Test, WMS-R, CPT, WAIS-R, FSIQ, SAS, AIMS, MANOVAs	Favor of adjuvant celecoxib
Asadabadi et al. (21)	Autism, *n* = 40	Double-blind, randomized, placebo-controlled add-on trial	Celecoxib 300 mg BID + Risperidone VS Placebo + Risperidone	Autism Behaviour Checklist Community Edition (ABC-C)Rating Scale (Irritability subsection)	Favor of adjuvant celecoxib

### The Link Between Inflammatory System, the Brain, and Mental Illness

In 1927, Julius Wagner-Jauregg became the first and the only psychiatrist thus far to win a Nobel Prize in Medicine. His impactful discovery involved the association with inflammation *via* malaria inoculation to cure neuropsychiatric symptoms of syphilis (22). Unfortunately, this inflammatory etiological theory was set aside during the advent of the psychotropic revolution (23). While support for the monoamine hypothesis in neuropsychiatric disorders continued to gain traction in subsequent decades, a residual group of patients exhibited persistent treatment-refractory illnesses and chronic debilitating symptoms suggestive of alternate hypotheses for neuropsychiatric conditions (3).

#### Innate and Adaptive Immunity

Immune system responses are typically classified as either innate or adaptive. The innate immune system features elements that are both genetically heritable and evolutionarily ancient, found in all multicellular organisms (24,). The innate system’s principal phagocytes include neutrophils, monocytes, and macrophages, which work in synergy to establish the first-line barrier of immunity (26). This line of defense is supplemented by the adaptive immune system, which includes specialized cells, B-lymphocytes and T-lymphocytes. Both response sectors produce a composite operation of moderating immunotransmitters, defined by cytokines. These immunomodulatory cytokines are typically categorized as pro-inflammatory or anti-inflammatory on the basis of their general effects. Pro-inflammatory cytokines such as tumor necrosis factor alpha (TNFα), interferon gamma (IFNγ), and interleukin (IL)-1 and IL-6 are primarily secreted by monocytes and macrophages, promoting additional complex inflammatory response systems, discussed in detail elsewhere [e.g., Ref. (27)]. Anti-inflammatory cytokines include IL-4, IL-10, IL-11, and IL-13 (26,). In simplistic terms, imbalanced pro-inflammatory over anti-inflammatory cytokine load will preferentially increase the throughput of pathological cellular pathways.

#### Central Nervous System Immunity

The blood–brain barrier is the brain’s primary defense against chemical insult. During peripheral inflammatory activation, there is increased permeability of the blood–brain barrier (29,). Such increases in blood–brain permeability may exacerbate or possibly even initiate neuropsychiatric and neurological disorders [see Ref. (31) for a review]. Furthermore, recent identification of lymphatic vessels within the central nervous system (CNS) reveals an alternate route of communication with the immune system to the brain (32). Once activated, a host of cellular and chemical pathways within the brain can result in significant structural change.

Microglia are specialized macrophages localized to the CNS that also play an important regulatory role in inflammatory response. They secrete neurotrophic factors important for cellular repair and signal recruitment for immune cells (26,,). The role microglia play in inflammation-driven neuronal damage and degeneration is also well established (35). Microglia have been shown to express different phenotypes or polarizations, classified as M1 and M2. M1 polarization is influenced by the pro-inflammatory state and acts in neuronal apoptosis, while the M2 polarization, in contrast, promotes neurogenesis (32,–39). Interferon alpha (IFNα) has been shown to induce a pro-inflammatory shift in microglial phenotype, from M2 to M1, resulting in depressive symptoms in mice (40,).

#### Inflammation and Neuropsychiatric Symptoms

When anti-inflammatory regulators are unable to balance pro-inflammatory reactions, inflammation can persist, in conjunction with sub-threshold neuropsychiatric symptoms (42). The following mechanisms describe the cytokine communication with neuropsychiatric symptoms and thus support the inflammatory hypothesis.

Pro-inflammatory markers have been associated with the development of neuropsychiatric symptoms (43). IL-6 activates the type 2 immune response, prompting the B-cell maturation pathway, consequently producing antibodies directed against extracellular pathogens. In addition, IL-6 activates the release of C-reactive protein (CRP) from the liver. Elevated levels of CRP and IL-6 in childhood were associated with an increased risk of developing depressive and psychotic symptoms in the future (44). Consistent with this, a significant association has been reported between CRP and several neuropsychiatric disorders, including depression, anxiety, and schizophrenia (45,). CRP is a well-established biomarker for an active inflammatory process and is a significant independent predictor of coronary heart disease risk (47–53). Furthermore, elevated CRP and IL-6 levels have also been associated with cognitive dysfunction (54). Studies have shown that biomarkers such as CRP and IL-6 may shed light on subtyping depression (55,). Several meta-analyses have demonstrated significant evidence of elevated pro-inflammatory cytokines in patients with depressive symptoms (43,,), bipolar disorder (58–60), schizophrenia (58,), obsessive compulsive disorder (OCD) (62), and autism spectrum disorders (ASDs) (63). These pro-inflammatory markers include CRP, IL-6, TNFα, and the IL-1 receptor antagonist (IL-1Ra) (43, 57–63). In addition, pro-inflammatory cytokines have been shown to trend toward normalization with symptom improvement indicating treatment response (64). Participation from microglia and peripheral macrophages are identified in activated inflammatory networks (65). An exaggerated immune response can be responsible for neuronal damage and decreased brain derived neurotrophic factor (BDNF), a protein integral to neuronal growth, plasticity, and survival (66–70). Excessive activation of the immune system may exacerbate mental illness in a subgroup of vulnerable individuals (71, 72). The inflammatory hypothesis suggests that hyperactivation of the immune system may produce nitro-oxidative stress and alterations of the kynurenine pathway, subsequently dysregulating monoamine levels and activating the glutamatergic system (2, 15, 73).

The evidence for cytokine-induced neuropsychiatric symptoms in healthy participants favors the inflammatory model. For example, healthy participants received an infusion of endotoxin to induce an inflammatory response, with resultant mood symptoms (74). Similarly, healthy individuals who received exogenous cytokines (IL-2, IFNα, and TNFα) also developed neuropsychiatric symptoms, including depression, mania, emotional dysregulation, cognitive impairment, and/or avolition (75). Elevated serum pro-inflammatory markers, TNFα, IL-6, and cortisol levels were observed by *Salmonella abortus equi* endotoxin injections (76). Subsequently, the subjects also exhibited neuropsychiatric symptoms of appetite changes, mood and anxiety symptoms, and cognitive decline without physical sickness symptoms (77). These findings were replicated with other vaccinations of healthy individuals (74, 78). Preclinical research has yielded similar findings, wherein lipopolysaccharide (LPS) and IL-1 injections in mice were found to result in sickness behavior, an analog to depressive symptoms (79).

Notably, pro-inflammatory agents such as recombinant IFNα and IL-2 have been used in the treatment of hepatitis C and carcinomas, respectively (80, 81). Interestingly, while effective for treating the targeted indication, IFNα was found to induce significant neuropsychiatric side effects with up to 80% of patients endorsing mild to moderate depressive symptoms (82–84). Grigoleit et al. (85) identified a positive dose-dependent association in IL-6, IL-10, TNFα, cortisol, and norepinephrine with neuropsychiatric symptoms. While some studies have reported that serum IL-6 and TNFα also appeared elevated in OCD patients compared to healthy controls [e.g., Ref. (86)], it should be noted that an earlier meta-analysis of OCD patients revealed no significant difference in TNFα or IL-6 (62). However, these authors did note a reduced level of pro-inflammatory IL-1β in OCD patients (62).

#### Autoimmune Conditions and Neuropsychiatric Disorders

Autoimmune and infectious conditions such as rheumatoid arthritis (87), type 1 diabetes (88), systemic lupus erythematosus (89), hepatitis, and sepsis (90) increase the risk of neuropsychiatric symptoms. An extensive Danish-based study found a 62% increased risk of mood disorders after infection-related hospitalizations (90). In patients who suffer from both Crohn’s disease and depression, exacerbations of both physical and mental illnesses tend to occur at the same time (91). Patients experiencing psoriasis and anxiety symptoms were shown to benefit from treatment with cytokine inhibitors (92–94). In addition, a greater prevalence of autoimmune conditions such as pemphigus in bipolar disorder is observed (95). In a large epidemiological study, multiple infections and autoimmune disorders were associated with the increased lifetime prevalence of schizophrenia spectrum disorders (96).

In summary, multiple studies have indicated a positive association between inflammation and neuropsychiatric symptoms. Elevated pro-inflammatory markers are consistently associated with neuropsychiatric symptoms and reveal a bidirectional relationship.

### Cyclooxygenase-2 (COX-2) Inhibitors

**Figure 1:**
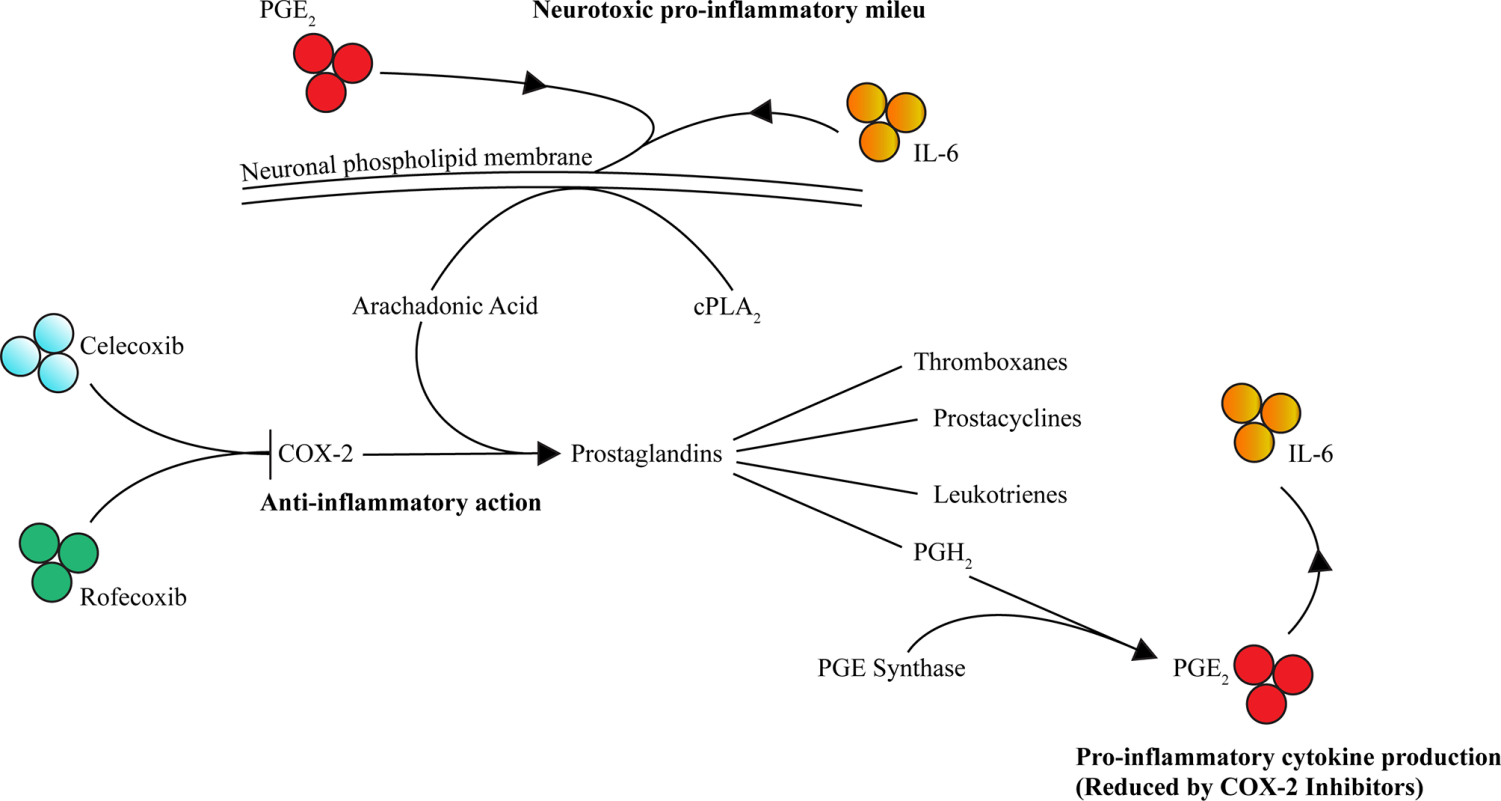
COX-2 contributes to inflammation through PGE_2_ and IL-6 and is selectively inhibited by celecoxib and rofecoxib. Activated by inflammatory cytokines including PGE_2_ and IL-6, AA (the precursor substrate of the COX-2 pathway) is extracted from phospholipid membranes by phospholipases such as cPLA2. COX-2 then drives production of prostaglandins including prostaglandin H_2_ (PGH_2_), which in turn is converted to PGE_2_ *via* PGE synthase. An accumulation of PGE_2_ leads to increased IL-6 (along with other cytokines) contributing to the inflammatory milieu, further potentiation of the pathway and neurotoxicity contributing to psychopathology. Celecoxib and rofecoxib exert selective inhibition of COX-2 reducing this pathway’s contribution to inflammation mediated neurotoxicity (→ = activates/increases, ⊥ = inhibits).

The COX pathway involves the precursor substrate of arachidonic acid (AA) to produce thromboxane, prostacyclin, and prostaglandins (PG) D2, E2, F2, and I2. AA is extracted from cell membranes by phospholipases, predominately cytoplasmic phospholipase A2 (cPLA2), and metabolized by the COX enzymes. The two rate-limiting enzymes within the COX pathways are COX-1 and COX-2. COX-1 is constitutionally expressed, and thus responsible for baseline prostaglandin levels, whereas COX-2 is inducible and expressed exclusively in the CNS, kidney, thymus, GI tract, and possibly in the female reproductive system (97–100). However, during inflammatory processes, COX-2 expression is promoted by and regulated by inflammatory stimuli, including lipopolysaccharide (LPS), IL-1, IL-6, TNF, IFNγ, and AA (101–108). The variability of isotype expressions COX-1 and COX-2 might be explained by the target tissue and type of insult (109, 110). The COX-2 enzyme produces prostaglandins, thromboxanes, prostacyclins, and leukotrienes downstream, which are suspected to be culprits for inflammation and neoplastic growth, in particular PGE_2_ (111). Furthermore, COX-2 expression is supported by microglia (112) and is auto-regulated *via* its by-products, PGE_2_ and PGF_2_α, perpetuating the inflammation process (113, 114). Additionally, PGE_2_ promotes IL-6 production (115), subsequently reinforcing this positive inflammatory feedback loop (**Figure 1**). Notably, in COX-2 gene knockout mice, a decrease in PGE_2_ and nuclear factor (NF)-κB activity was observed (114, 116, 117).

In 1995, the first generation of selective COX-2 inhibitors, celecoxib and rofecoxib, entered clinical trials (118). Over the next 4 years, numerous trials demonstrated selective COX-2 inhibitors could reduce pain and inflammation (119). From 2000 to 2004, larger trials such as CLASS, VIGOR, and TARGET identified the reduced gastrointestinal risks associated with COX-2 inhibitors, however, highlighted the increased cardiovascular risks leading to rofecoxib being pulled from the market (120–123). In 2005, valdecoxib was also withdrawn from the market for similar concerns by the Food and Drug Administration (FDA). In 2011, celecoxib was withdrawn from the market for the indication of cancer prevention while still being indicated for rheumatoid arthritis, osteoarthritis, and acute pain. Subsequently, this led to cautious prescribing due to concerns of cardiovascular side effects (124).

Preclinical trials have shown that rofecoxib can increase serotonin levels in the frontal and temporoparietal cortex (125). In addition, celecoxib was shown to potentiate the effects of reboxetine and fluoxetine on cortical noradrenaline and serotonin output (126).

Although ubiquitously expressed, COX-1 is typically described as gastro-protective and neuroprotective (127, 128), and pre-clinical data reveal some contradictory evidence regarding the inflammatory classifications of the COX isoforms. COX-2 may have anti-inflammatory and neuroprotective properties, aiding in neurotransmitter release, long-term potentiation, blood flow regulation, and memory consolidation (129). It was reported that COX-2 gene knockout mice were susceptible to inflammation compared to healthy mice (130, 131). Deletion of the COX-2 gene causes increased permeability of the blood–brain barrier and leukocyte infiltration (132). Another study showed that maximal COX-2 expression coincided with inflammatory resolution and was associated with minimal PGE_2_ synthesis (133).

Nevertheless, COX-2 inhibitors have proven to be beneficial in glutamate-mediated death prevention and suppression of pro-inflammatory cytokines (134, 135). Kainic acid (KA), a potent neurotoxin, elicits excitatory effects on *N*-methyl-D-aspartate (NMDA) receptors resulting in status epilepticus, neurodegeneration, and memory loss (134–138). KA has also been shown to increase COX-2 expression in the CNS (139); therefore, COX-2 inhibitors have been shown to prevent KA-induced neuronal death (140). There appears to be mounting evidence of therapeutic effect of COX-2 inhibitors in mediating glutamatergic processes (141–143). As well, there seems to be appropriate evidence for COX pathway in neuropsychiatric disorders.

### Pathophysiology of Neuropsychiatric Disorders and the Efficacy of Cyclooxygenase-2 Inhibitors

We have now established there is substantive evidence for inflammation being a driver of neuropsychiatric symptoms by negatively impacting on neuronal proliferation, survival, and differentiation (144, 145). Furthermore, the COX pathway potentiates the inflammatory process and may exacerbate inflammation mediated neurodegeneration. COX-2 inhibition reduces this inflammatory load and thus the impact of these pathways on the brain.

In this section, we address the proposed mechanisms by which inflammatory states influence central monoamine effects, the hypothalamic–pituitary–adrenal (HPA) axis, and microglial activation, pathways at the center of neuropsychiatric pathogenesis. Discussion will focus on specific conditions including depression, bipolar disorder, schizophrenia, ASD, and OCD. Herein, a number of clinical trials investigating the efficacy of the cyclooxygenase-2 inhibitors for neuropsychiatric disorders are discussed, summarized in **Table 1**. Particular attention is being paid to the operative inflammatory pathways inherent to these conditions and the potential role for COX-2 inhibitors in their management.

#### Neurotransmitter Dysregulation Hypothesis

The dysregulation of the neurotransmitters such as serotonin, norepinephrine, dopamine, acetylcholine, and glutamate has been the foci of the biochemical etiology of neuropsychiatric illnesses. While treatment with antidepressants and neuroleptics aims to modulate monoamine signaling, there is a wealth of evidence supporting secondary mechanisms of action including effects on inflammatory pathways (27, 146). During inflammation, the pro-inflammatory cytokines IL-2 and IFNα have been shown to directly increase enzyme activity of the indoleamine-pyrrole 2,3-dioxygenase (IDO) enzyme of the kynurenine pathway, which promotes conversion of tryptophan to kynurenine, consequently depleting the antecedent supply to serotonin (147), in addition to direct catabolism of serotonin by IL-6 and IFNα (148, 149). This evidence supports the monoamine hypothesis regarding the hypoactive serotonin state featured in mood disorders (150–152). IFNα administration led to inflammation by increasing the concentration of kynurenine pathway metabolites in the CSF, namely kynurenine, kynurenic acid, and quinolinic acid (QUIN) (153). These metabolites have been presented as inducers of depressive and anxiety symptoms (154). Notably, QUIN can selectively activate NMDA receptors (155, 156) and has been associated with numerous neurological diseases, including: Alzheimer’s disease, anxiety, depression, epilepsy, human immunodeficiency virus-associated neurocognitive disorders, and Huntington’s disease (155, 157–161). QUIN has also been shown to cause neurodegeneration *via* multiple models (159).

#### HPA Axis Dysregulation

During a stress response, the HPA axis is activated (162). The hypothalamus secretes two hormones, corticotrophin releasing hormone (CRH) and arginine vasopressin, which act on the pituitary gland to increase adrenocorticotropic hormone (ACTH) release, subsequently accelerating the production of cortisol to aid in the homeostasis feedback loop (163). Studies have demonstrated an elevated inflammation state perpetuating cytokines such as IL-1, IL-6, TNFα, and IFNα; these in turn activate the HPA axis elevating levels of CRH, ACTH, and cortisol (162, 164–167). This relationship furthermore supports the feedback loop maintaining a hyperactive HPA system (163). Chronic elevation of endogenous glucocorticoids results in mood symptoms (163, 167). Additionally, it is proposed that cortisol increases the catabolizing enzyme tryptophan 2,3-dioxygenase (TDO) to deplete the precursor to serotonin implicating an association with the serotonin dysregulation (168, 169).

Contrary to advantageous effects of steroids in managing infections, there is evidence that glucocorticoid treatment duration for acute infections versus chronic infections results in changes of glucocorticoid receptor function and concentrations (170, 171). This subsequently influences HPA axis hyperactivity, which elevates cortisol and results in decreased function and quantity of glucocorticoid receptors resulting in impaired feedback and glucocorticoid resistance (170). Glucocorticoid resistance that is seen in depressed patients may also be a result of changes in expressed glucocorticoid receptors ratio *via* cellular phosphorylation (172–174). Patients with neuropsychiatric illnesses also exhibited heightened plasma, urine, and cerebrospinal fluid (CSF) levels of cortisol and anatomical changes in the pituitary and adrenal glands (175–178). Pavon et al. (179) reported elevated cortisol levels in depressed patients associated with elevated TNFα, in addition to decreased levels of IL-1β, suggesting that increased cortisol may influence inflammatory cytokines. However, it is important to bear in mind that while some subtypes of depression (namely melancholic or endogenous) are associated with hyperactive HPA axis, glucocorticoid resistance, and increased circulating cortisol levels, atypical and seasonal depression has been consistently reported to have normal or hypoactive HPA axis function (180, 181). Therefore, the hypothesis is supported regarding the impairment of the HPA axis through cellular mechanisms and dysfunctional feedback leading to HPA axis dysfunction, one of the most consistent findings in biological psychiatry, which is exhibited by patients with depression, bipolar disorder, and schizophrenia (169, 182).

#### Microglial Hyperactivation Hypothesis

Microglia function as macrophages of the CNS by clearing foreign particles and promoting healing after traumatic brain injury (183). They are also involved in the pruning process of neurons by tagging unutilized synapses for degradation to rebuild more active neurons during the maturation process (184). Pathological synaptic pruning may also contribute to prodromal, remittent and relapsing, and chronic stages of neuropsychiatric disorders (185–187). Prolonged microglial activation induces synaptic pruning subsequent to the accumulation of two pro-inflammatory cytokines, specifically TNFα and IL-1β, leading to neuronal apoptosis (188, 189). The subsequent dysfunctional neuronal pathways may be compensated by adaptive systems, which may resultantly produce and preserve maladaptive behaviors (190, 191). Individuals at an ultra-high risk for developing schizophrenia also appear to have significantly elevated activity of microglia (192). Histological changes in activated microglia have been observed in patients with schizophrenia who had committed suicide during an acute episode of psychosis (193). In contrast, conflicting data derived from post-mortem studies have reported reductions in microglial density and activation (194). These findings may indicate a difference of microglial activation depending on the stage of illness among other factors (194). Aberrant microglial activation is seen in other neuropsychiatric disorders including Alzheimer’s dementia, Parkinson’s disease, multiple sclerosis, herpes encephalitis, traumatic brain injury, and stroke (195, 196). Alterations in brain morphology have been described across the spectrum on neuropsychiatric conditions (197–201). Duration of mental illnesses also has evidence of significant brain morphologic changes (201). Mechanisms that may encourage these anatomical reductions include oxidative and nitrosative stress through activation of microglia (202).

### Depression

Major depressive disorder (MDD) is highly prevalent throughout the world, and the prevalence has increased over time (73). The estimated lifetime prevalence of major depression and persistent depressive disorder in adults is 12% (203). In unipolar depression, inflammation and depressive symptoms share a bidirectional relationship. Immunological markers such as CRP, and cytokines IL-1, IL-6, and TNFα, are elevated in patients with depression (1, 204, 205). A recent meta-analysis comprised of 3,212 participants noted elevations in the concentrations of IL-6, TNFα, IL-10, the soluble IL-2 receptor, C-C chemokine ligand 2, IL-13, IL-18, IL-12, IL-1 receptor antagonist, and the soluble TNF receptor 2 in depression patients (1). Several groups of authors have reported that IL-6 may be a useful biomarker for predicting treatment response (115, 204–207). A prospective study revealed a correlation with elevated serum IL-6, as those with higher levels were more likely to be depressed by 18 years of age than individuals on the lower end levels (208). Pro-inflammatory cytokines have been shown to trend toward normalization with symptom improvement indicating treatment response (64).

Studies have shown biomarkers such as CRP and IL-6 may shed light on depression subtypes (55, 56). An interesting larger scale study, The Netherlands Study of Depression and Anxiety (NESDA), extrapolated gender variance while evaluating CRP and IL-6 (181). The authors described an increased level of CRP and IL-6 with normal levels of TNFα in male patients with depressive symptoms; however, there were no associations with the cytokines in women with depressive symptoms (209). Additionally, they noted a differential role of the HPA function, inflammatory markers, and metabolic variables between melancholic and atypical depression subtypes (181).

Elevated levels of kynurenine pathway toxic metabolites such as QUIN are also observed in patients with depression (210). Interestingly, in a 6-week RCT, Krause et al. (211) noted a correlation with kynurenine/tryptophan ratios that was predictive of celecoxib response to significant improvements in the Hamilton Depression Scale (HAMD-17) scores. Subsequently, the kynurenine/tryptophan ratio shows some promise as a potential biomarker for predicting response to COX-2 inhibitors.

Gałecki et al. (212) reported an increase of non-coding micro ribonucleic acid (mRNA) expression of the COX-2 enzyme in recurrent depression. COX-2 inhibitors decrease IDO activity, subsequently decreasing glutamatergic-active by-products such as QUIN, which may add in neuro-stabilizing effects (4, 213, 214). Higher concentrations of QUIN and 3-hydroxykynurenine have been reported in depression (210). Preclinical studies have shown that celecoxib administration in rats was associated with reductions in PGE_2_ levels and a reversal of stress-induced depressive-like behaviors (215, 216). PGE_2_ had been shown to contribute to monoamine imbalance with decreased norepinephrine central neuronal release and dysregulation of the HPA axis (217). Consequently, this alters cortisol synthesis and subsequently suppresses serotonin (215, 218–220). Consistent with this assertion, an animal-based model of depression in rats demonstrated that celecoxib independently enhances the release of serotonin in the brain (126).

Celecoxib has also been shown to attenuate pro-inflammatory cytokines IL-1β and TNFα and significantly increase IL-10 levels in animal models (109, 221). IL-1β has been identified as a modulator of BDNF (144). Some evidence exists indicating that elevated levels of IL-6 results in a reduction in BDNF, implicating in imbalanced neurogenesis, resulting in neural circuitry dysfunction in depressive symptomology (144). In a preclinical study with rats with a depression-like phenotype, augmentation with acetylsalicylic acid, a non-selective COX inhibitor, enhanced the efficacy of fluoxetine (222).

To date, several reviews have suggested that celecoxib may be efficacious for the management of depressive symptoms (1, 223, 224); however, some have suggested it is clinically inadequate (225). Abbasi et al. assigned adjuvant celecoxib to MDD patients on sertraline and measured IL-6 levels in samples of their serum. They reported a correlation between decreasing IL-6 concentrations and improvement in the Hamilton Depression Rating Scale (HDRS) scores as outlined in **Table 1**, along with the key findings of all clinical trials assessing celecoxib in neuropsychiatric disorders appearing in this review. In another RCT (8) assessing celecoxib augmentation with sertraline in the treatment of drug-naïve women with depression, the authors reported an improvement in HDRS and Hamilton Anxiety Rating Scale (HAM-A) scores compared to the placebo group after 4 weeks of treatment. However, there were no significant differences between both groups at the end of the 8-week trial. Interestingly, the remission rates in the celecoxib group were statistically higher in comparison to the placebo group. Subsequently, Akhondzadeh et al. assessed the HDRS in 40 individuals in a 6-week RCT receiving fluoxetine plus celecoxib versus fluoxetine alone. They demonstrated significant improvements in depressive symptoms, and response and remission rates in the celecoxib group (5). Another RCT found more significant improvements in depressive symptoms in the adjuvant celecoxib group with reboxetine compared to reboxetine alone (4). However, Fields commented that there were no significant changes in late-life depressive symptoms in patients prescribed either placebo, celecoxib, or naproxen (6). These discrepancies in the efficacy of COX-2 inhibitors for this indication might be explained by methodological heterogeneity and variance in target sample characteristics. For example, the Geriatric Depression Scale (GDS) was used for the geriatric patients with a yearly frequency, which may not be a specific tool for detecting variations in depression diagnosis (226). However, the study did have strength in having a large sample size and median follow-up of 2 years with patients.

Inflammation may be the primary mechanism of pathogenesis in brucellosis (227, 228). Therefore, Jafari et al. assessed 40 individuals with celecoxib for treatment of mild to moderate depression due to acute brucellosis. They reported an improvement of the HDRS in the 8-week trial with the celecoxib with antibiotics group than placebo with antibiotics (9).

In patients with comorbid osteoarthritis, pooled data from five post-approval trials, each at 6 weeks in length, participants were randomized in placebo, ibuprofen or naproxen, or celecoxib groups while assessing the Patient Health Questionnaire-9 (PHQ-9). The authors report a trend toward a reduction in PHQ-9 depression scores. However, this lack of robust data is possibly due to the lack of efficacious dosing of celecoxib of 200 mg (229).

Interestingly, celecoxib may exhibit benefits in patients with colorectal cancer. Investigations have illustrated that celecoxib initiation in the head, neck, and gastrointestinal cancer population is associated with improvements in biological symptoms of depression, including an increase in appetite, body mass index, and quality of life (230, 231). A 6-week RCT included 40 colon cancer participants randomly assigned to either celecoxib monotherapy or a placebo group, which resulted in significant improvements in the HDRS among the former group, starting as early as week 2 and was sustained until the end of the trial (11).

Another cancer trial consisted of 52 outpatients with breast cancer undergoing 6 weeks of treatment with either celecoxib or diclofenac for mild to moderate depression. The outcome measures were scored using the HDRS to compare the COX-2 inhibitor with an indiscriminate COX-inhibitor. They reported significant improvements in depressive symptoms in both groups by week 3 and significantly more considerable improvements with the celecoxib group compared to diclofenac by week 6. None of the participants experienced remission HDRS less than or equal to 7 (10). A meta-analysis demonstrated with 150 participants showed that the adjunctive celecoxib cohort had better response rates and remissions compared to placebo (224). In summary, interactions between the immune system and neurotransmitters, the tryptophan/kynurenine system, and the glutamatergic system provide links between the immune system and depression; furthermore, data are suggestive of a role for celecoxib in treatment of depressive symptoms (115).

### Bipolar Disorder

The estimated lifetime prevalence of bipolar disorder among adults worldwide is 1% to 3% (232). For many, bipolar disorder is a chronic and debilitating illness, with patients often experiencing poor inter-episodic remission (233, 234). Pro-inflammatory markers, such as IL-4, TNFα, IL-1β, and CCL2 cytokine, which have an established role of inflammation in neuronal damage and degeneration, have been observed to be elevated in patients with bipolar disorder (35, 59, 233–236). Elevated CRP levels were also identified in a meta-analysis of 730 patients with bipolar disorder (237).

Interestingly, during the euthymic phase of bipolar disorder, IL-4 has been shown to return to baseline levels; this apparent relationship between inflammatory and mood states provides an avenue for prospective biomarker investigations (65, 238). Furthermore, accumulating evidence is suggestive of chronic low-grade inflammation in bipolar patients (239, 240). Scans employing positron emission tomography (PET) have supported neuroanatomical changes and hyperactive microglial state in bipolar disorder (241–244). Gray matter reduction was observed in the anterior limbic region (197, 198) including ventricular enlargement (245). Lithium, a well-established mood stabilizer, has been shown to reduce IL-2, IL-6, IL-10, and IFNα levels after long-term use, possibly inferring its nebulous mechanism of action through anti-inflammatory processes (246). In addition, lithium has some potential in neurogenesis, which may be linked with particular anti-inflammatory mechanisms (202). Preclinical data also suggest that these mood stabilizing effects may downregulate the AA cascade, therefore decreasing COX-2 and prostaglandin levels (247–253).

A clinical trial investigated the efficacy of celecoxib in bipolar depression or mixed episode and found lower HAM-D scores initially after 1 week; however, no statistically significant difference was found at the end of the 6-week trial (12). In another study, celecoxib augmentation was trialed in individuals with acute mania without psychotic features alongside treatment-as-usual. This 6-week RCT demonstrated that adjuvant celecoxib with valproate was significantly effective for treatment in acute mania compared to valproate and placebo (14). The difference in trial outcomes may suggest greater inflammatory impact and therefore COX-2 inhibitor efficacy during the manic phase of illness, as opposed to the depressive phase; this is also supported by the relative increase in inflammatory markers in the former illness phase (66).

Electroconvulsive therapy (ECT) is an effective treatment modality for various phases of illness in bipolar disorder (254). ECT is reported to affect monoamines, hormones, in addition to the immune system, cytokines, ACTH, and cortisol (255–257). Immunomodulatory effects have also been reported, for example, effects on the kynurenine pathway *via* the decrease of QUIN concentrations in unipolar and bipolar depression (258, 259).

Kargar et al. (260) assessed cytokine and CRP changes in patients receiving both ECT and celecoxib, reporting a reduction in TNFα, but no significant changes in other inflammatory markers, such as IL-1β, IL-6, and CRP. However, they noted greater clinical improvement of depressive symptoms in the first week of celecoxib intervention but no persisting differences thereafter (260). Notably, the authors hypothesized that immunomodulatory effects associated with ECT might explain the baseline return of TNFα concentrations; however, no significant cytokine changes were observed. This may also be due to the post-ECT acute induction of cytokines hindering statistical significance (255, 261). Another RCT with 35 ECT participants with focus on BDNF levels in patients with mania concluded no statistical difference in BDNF levels or treatment efficacy with adjuvant celecoxib (13). The authors of this study suggested these effects were nearing statistical significance, and that their BDNF sampling protocol may have been a confounding factor (13). A longer multicenter trial has been proposed to assess augmentation with celecoxib and/or minocycline alongside treatment as usual (TAU) in bipolar I or bipolar II patients in depressive state; however, these results are yet to be published (262). Bipolar disorder has significant associations with inflammatory modulation resulting in aberrant brain changes that warrant further investigation of the roles of anti-inflammatory agents. In particular, celecoxib shows some promise requiring further investigation, particularly during a set phase of the bipolar illness.

### Schizophrenia

Schizophrenia is a severe, chronic, and among the most disabling and economically catastrophic medical disorders. The World Health Organization ranks schizophrenia as one of the top 10 illnesses contributing to the global burden of disease (263). The dysregulation of dopamine and glutamatergic pathways in various brain regions are implicated in the positive, negative, and cognitive symptom domains of schizophrenia (264). Pro-inflammatory cytokines such as IL-1β and IL-6 can influence neuronal development, specifically on the dopaminergic and serotonergic systems (265–268). IL-1β administration after birth can influence the dopamine system in adulthood, which has been associated in dopaminergic and serotonergic neuronal moderation in rat models (266). In schizophrenia, elevated serum and CSF concentrations of kynurenine were reported (269). Developing findings are suggesting that infectious exposure during the prenatal period may contribute to the pathogenesis of schizophrenia (270). Raised maternal levels of IL-8 during pregnancy are associated with an increased risk for schizophrenia in offspring, in addition to decreased brain volumes, independent of the inflammatory etiology (271). Maternal immune activation in animal models generated oxidative stress in the fetus (264). Observed infectious agents including *Toxoplasma gondii*, Chlamydia, bornavirus cytomegalovirus, and influenza seem to increase the risk of schizophrenia. This may, however, occur as a result of the immune response rather than an infectious etiology (270). Several epidemiological studies have observed an elevated prevalence of schizophrenia in cohorts born during influenza epidemics (272) and significant association with immunological disorders (273). There is a higher prevalence of schizophrenia in individuals with celiac disease, bullous pemphigoid, interstitial cystitis, thyrotoxicosis, and acquired hemolytic anemia (273, 274). Surprisingly, rheumatoid arthritis reveals lower rates of co-morbid schizophrenia compared to the general population (275).

Post-mortem brain studies from schizophrenia patients have revealed significant inflammatory processes (276). In addition, PET imaging has signified microglial activation resulting in brain morphological changes in first-episode and chronic psychosis (277–280). These morphological changes have been expressed during prodromal phases of first-episode psychosis (281–283), suggestive of neurotoxic processes resulting in poor prognosis (281, 284). Moreover, some authors have shown a relationship between brain volume, IL-1, and IL-6 (285–287). Collectin inflammatory markers have also been implicated in patients with schizophrenia, C4A in particular, whose role is to influence microglial hyperactivity, neurodegeneration, and subsequent brain cortical volume reductions (288, 289). These microglial changes may derive from established elevations in serum pro-inflammatory factors, such as PGE_2_, CRP, IL-1β, IL- 6, IL-8, and TNFα (290–292). In addition, cytokines have shown some correlation with negative symptoms, cognitive deficits, and psychomotor retardation (293–295).

Positive correlations with cognitive severity and inflammatory markers have also been highlighted (296–298). In the first-episode and acutely relapsed patients with psychosis, an elevated level of pro-inflammatory cytokines, IL-6, TNFα, TGF-β, and IFNγ, was observed (61). In addition, IL-10 concentrations were decreased in acutely relapsed patients compared to controls (61). Cytokine concentrations at different stages of the disease and variable treatment agents may alter neuroprogression (115, 299).

Further support is provided from studies suggesting antipsychotics may exhibit immune-modulatory properties (264); however, there remains evidence that is contrary to this notion (300, 301). Patients undergoing long-term treatment with antipsychotics exhibit reduced pro-inflammatory cytokine levels (IL-1β, IL-6, sIL-6R, and TNFα) (302–304) and elevated anti-inflammatory markers (sIL-1RA, sIL-2R, and IL-10) (305–307). Second-generation antipsychotics may elicit more potent anti-inflammatory effects than to first-generation agents (303, 308).

Dysregulation of the tryptophan metabolism has been implicated with notable elevations of kynurenic acid in patients with schizophrenia (309). Furthermore, long-term antipsychotic treatments have an impact on kynurenic acid concentrations in rodent models (310). Preclinical studies suggest that COX-2 inhibitors protect against glutamate-mediated neurotoxicity, which may highlight an application to mediate neurodegeneration in kynurenine system (18, 135, 311).

One of the first RCTs evaluating the use of COX inhibitors for schizophrenia indication consisted of 50 patients with acute exacerbation of psychosis who were admitted and treated with risperidone with one group augmented with celecoxib versus placebo for 5 weeks (17). The celecoxib group revealed significant positive effects on the Positive and Negative Syndrome Scale (PANSS) (17). *Post hoc* analyses indicated an improvement in cognition parameters with augmentation of celecoxib in schizophrenia in this trial (312).

In contrast, Rapaport and colleagues assessed outpatients with schizophrenia on stable psychotropic regimens of olanzapine or risperidone, finding no significant changes with celecoxib augmentation in several of the psychometric parameters (18). This finding could be explained by differences in the study cohorts, given the participants in Müller’s study were acutely psychotic, whereas Rapaport’s sample consisted of patients in more stabilized psychotic states.

During an 8-week RCT, the treatment of 60 acutely psychotic patients was augmented with celecoxib (19). The risperidone and celecoxib combination was superior in the improvement of PANSS total scores over risperidone alone (19). Also, the Extrapyramidal Symptoms Rating Scale (ESRS) scores for the placebo group were higher than in the celecoxib group over the trial but not statistically significant (18). Müller et al. (20) completed a 6-week, RCT of 49 patients during their first-episode of schizophrenia. They were treated with amisulpride with random assignment of celecoxib or placebo (20). There was an improvement in the PANSS in the adjunct celecoxib group compared to the placebo group (20). The adjunct celecoxib group in this study also showed a significant improvement on the clinical global impression (CGI) scale (20). Overall, a superior therapeutic effect with augmentation with celecoxib was found, in particular a trend for improvement in negative symptoms.

A recent meta-analysis, including the above RCTs in addition to two inaccessible RCTs, revealed that adjunctive celecoxib did not prove efficacy over placebo in overall samples (313). However, with the sub-analysis, they discovered superior efficacy with celecoxib to placebo in first-episode patients (313). This may be explained by data from preclinical studies suggesting celecoxib’s effects on cytokines and behavioral symptoms are dependent on the stage of illness and time of intervention (217).

It is important to note the comorbid conditions that may contribute to inflammation and confound interpretation of outcomes, including trauma, stress, smoking, metabolic syndrome, diet, exercise, and poor dental hygiene (2). Nonetheless, there is an apparent association between inflammation and schizophrenia, with celecoxib demonstrating promise possibly during early disease onset.

### Autism Spectrum Disorder

ASD is a neurodevelopmental disorder defined by impairments in two domains: 1) shortages in social communication and social interaction and 2) restricted repetitive patterns of behavior, interests, and activities (314). The prevalence of ASD in Western countries appears to have increased, possibly as a result of definition changes and heightened awareness. The pathogenesis of ASD remains idiopathic, although the consensus points to altered brain development leading to impairment in social and communication maturation, therefore resulting in restricted interests and repetitive behaviors (315). These brain morphologic aberrations appear to be a result of neural pruning processes and neuroinflammation (316–319).

An association with ASD and inflammatory response through the measles, mumps, and rubella (MMR) vaccine and enterocolitis were reported first in 1998 (320) and subsequently retracted. However, controversy still exists among these allegations in select groups, despite it being established that there is no causal association between MMR vaccine and ASD (320–324).

Similar to discussions in Schizophrenia section with respect to schizophrenia, prenatal infections during early development may also be associated with the development of ASD (325–327). Moreover, there seem to be shared immune-related genetic abnormalities between the two disorders (325, 328). Aberrant activity of the glutamatergic system might play a role in neurotoxicity of both disorders. Kynurenine pathway abnormalities may also be linked to 16p11.2 mutations in ASD resulting in glutamatergic activity (329).

Associations with changes in the immunomodulatory system of ASD patients have been identified. Disruption in immunomodulatory proponents such as T-cells and monocytes have been noted (316, 330), in addition to changes in the concentration of immunoglobulins (331) and autoantibodies production (332). Furthermore, polymorphisms identified in macrophage migration inhibitory factor (MIF), seen in ASD-related abnormalities, seem to also activate the COX-2 system in microglia (333, 334).

Post-mortem studies revealed greater microglial densities in the visual cortex, cerebellum, anterior cingulate gyri, and dorsolateral prefrontal cortex (DLPFC) of ASD patients (335–338). Some studies have also shown elevated TNFα, IFNγ, IL-1, IL-6, IL-8, IL-12, CCL2, CCL5, and CCL11 in plasma and CSF of autistic subjects (317, 318, 338–340).

A study showed that repurposed anti-inflammatory agents such as pioglitazone resulted in moderation of irritability, lethargy, stereotypy, and hyperactivity symptoms in ASD (341). The pioglitazone class of drugs has been shown to inhibit COX-2 in LPS-stimulated microglia and neurons (342, 343). Celecoxib in the rat model has shown to also inhibit LPS-induced neuronal toxicity (344).

Our search yielded only one randomized, double-blind placebo-controlled trial of celecoxib combination with risperidone. Asadabadi et al. (21) assessed 40 patients diagnosed with ASD in a 10-week trial with the aberrant behavior checklist-community edition (ABC-C), which showed superior efficacy of adjuvant celecoxib with risperidone in the domains of irritability, social withdrawal, and stereotypy in children with ASD.

Paucity of evidence is opportune for further investigations with COX-2 inhibitors in neurodevelopmental disorders such as ASD as seen by preclinical and limited clinical data.

### Obsessive-Compulsive Disorder

Obsessive-compulsive disorder (OCD) is a relatively common neuropsychiatric disorder with a reported lifetime prevalence of 1–3% in the general population (345, 346). At least one-third of individuals with OCD fail to adequately respond to current pharmacological treatment (347, 348). The cortico-striatal-thalamo-cortical (CSTC) circuit dysfunction is implicated in the pathophysiology of OCD (349).

There is evidence of early childhood infections, pediatric acute-onset neuropsychiatric syndrome (PANS), which encompasses pediatric autoimmune neuropsychiatric disorders associated with streptococcal (PANDAS), evoking OCD-like neuropsychiatric symptoms (15). This is suggestive of an inflammatory etiology to a subsect of this illness. Furthermore, immunomodulation treatment resulted in improvement of OCD-like neuropsychiatric symptoms (350).

Although the minority of cases of OCD results from PANS, it is speculated that the active inflammatory model may be relevant for the progression of OCD. As well, brain morphological changes have also been noted in OCD indicating progression in the neurodegenerative process (351). Rodent models exposed to LPS-induced inflammation exhibited increased anxiety with reduced exploration in the open field test (352, 353). The inducible chemokine, CXCL12, resulted in anxiety-like features in rat models (353), further supporting the role of inflammation in neuropsychiatric symptoms in anxiety disorders.

Animal models are suggestive of alternative microglial phenotypes, resulting in OCD-like behavior (354). Translocator protein distribution volume, a marker of increased microglial activation and thus neuroinflammation (355–358), was investigated after a prior study found increased expression in PANDAS patients (359). Kumar et al. discovered an increased translocator protein density in the CSTC circuit compared to healthy controls. Interestingly, this circuit involves multiple neuropsychiatric disorders aforementioned such as Huntington disease, cerebral vascular disease, Tourette disorders, and Sydenham chorea (360, 361). Repurposed microglial modulators such as minocycline have shown a reduction in the Yale-Brown Obsessive-Compulsive Scale (Y-BOCS) scores in a recent RCT in combination with fluvoxamine (362).

Most recently, Konuk et al. (86) reported significantly elevated levels of both IL-6 and TNFα in OCD patients compared to healthy controls. Furthermore, a correlation between elevated TNFα and onset with minimal association between IL-6 levels and duration of illness (86). A meta-analysis revealed no significant findings in TNFα and IL-6 plasma levels in OCD patients relative to controls; however, the authors did note reduced IL-1β in OCD patients (62). As celecoxib is shown to reduce levels of both IL-6 and TNFα, support for improving clinical symptoms of OCD is plausible (6).

Sayyah et al. (16) noted an improvement in the Y-BOCS for OCD patients with the augmentation of celecoxib with fluoxetine compared to fluoxetine alone. In addition, another recent RCT utilizing fluvoxamine with celecoxib augmentation compared to fluvoxamine alone noted an improvement in the celecoxib group (15). It is proposed that the notable efficacy in OCD, in addition to the microglial mechanisms, results from increased monoamines such as norepinephrine and serotonin *via* inhibition of prostaglandin synthesis by celecoxib (5, 306). However, further studies with larger sample sizes, longer duration, and measurements of pro-inflammatory markers may provide more robust evidence.

## Conclusion

Evidence for the inflammatory hypothesis and the role of anti-inflammatory agents continues to accumulate suggesting etiological impact on the development of neuropsychiatric conditions, such as depression, bipolar disorder, schizophrenia, ASD, and OCD. A promising body of evidence suggests a role for COX-2 inhibition, in particular celecoxib, for phase-related interventions in bipolar disorder, schizophrenia, and possibly depression, ASD, and OCD. Despite the paucity of data for COX-2 inhibitors and investigated agents, including aspirin, minocycline, and statins, with purported pleiotropic anti-inflammatory mechanisms, further research is necessary to clarify the role of immunomodulation therapies and their comparative efficacies for integration of the psychiatric professions’ current paradigm of treatment modalities (146, 363).

## Author Contributions

RS was the primary author of this manuscript, conducted initial literature search, review and synthesis of first draft and subsequent revisions. NG-C contributed to literature search and revisions. AW contributed to synthesis initial draft and revisions. OR contributed to manuscript draft, revisions and created figure. BA contributed to drafting and revision process. MB and SD were lead investigators providing initial concept and ongoing guidance throughout.

## Funding

Berk A is supported by Stanley Medical Research Foundation NIH, MBF, NHMRC, NHMRC Senior Principal Research Fellowship (APP1059660 and APP1156072), Cooperative Research Centre, Simons Autism Foundation, Cancer Council of Victoria, MBF, Rotary Health, Meat and Livestock Board Woolworths, BeyondBlue, Geelong Medical Research Foundation Bristol Myers Squibb, Eli Lilly, Glaxo SmithKline, Organon, Novartis, Mayne Pharma, Servier. Berk is the speaker in Astra Zeneca, Bristol Myers Squibb Eli Lilly, Glaxo SmithKline Lundbeck, Pfizer, Sanofi Synthelabo Servier, Solvay, Wyeth; and consultant in AstraZeneca, Bristol Myers Squibb Eli Lilly, Bioadvantex, Merck, Glaxo SmithKline Lundbeck, Janssen Cilag Servier. Berk is a co-inventor of two provisional patents regarding the use of NAC and related compounds for psychiatric indications, which, while assigned to the Mental Health Research Institute, could lead to personal remuneration upon a commercialisation event.Walker A is supported by the Trisno Family Fellowship.

## Conflict of Interest Statement

The authors declare that the research was conducted in the absence of any commercial or financial relationships that could be construed as a potential conflict of interest.

## Abbreviations

3MS-E, Modified Mini-Mental State Exam; AA, arachidonic acid; ABC-C, autism behavior checklist community edition; ACTH, adrenocorticotropic hormone; ASD, autism spectrum disorder; BDNF, brain derived neurotrophic factor; CCL2, C-C motif chemokine ligand 2; CGI-I, Clinical Global Impression: Improvement; CGI-S, Clinical Global Impression: Severity; CNS, central nervous system; COX, cyclooxygenase; CSF, cerebrospinal fluid; cPLA2, cytoplasmic phospholipase A2; CRH, corticotrophin releasing hormone; CRP, C-reactive protein; ECT, electroconvulsive therapy; ESRS, Extrapyramidal Symptoms Rating Scale; GDS, Geriatric Depression Scale; HAM-A, Hamilton Anxiety Rating Scale; HAM-D, Hamilton Depression Rating Scale; HDRS, Hamilton Depression Rating Scale; IDO, indolamine 2,3 dioxygenase; IL, interleukin; IL-1ra, interleukin-1 receptor antagonist; IFNα, interferon alpha; IFN-γ, interferon gamma; KA, kainic acid; LPS, lipopolysaccharide; MDD, major depressive disorder; mRNA, microribonucleic acid; NMDA, *N*-methyl-D-aspartate; NF, nuclear factor; OCD, obsessive compulsive disorder; PANS, pediatric acute-onset neuropsychiatric syndrome; PANDAS, pediatric autoimmune neuropsychiatric disorders associated with streptococcal; PANSS, Positive and Negative Syndrome Scale; PHQ-9, Patient Health Questionnaire-9; PGE2, prostaglandin E2; QUIN, quinolinic acid; RCT, randomized control trial; SANS, Scale for the Assessment of Negative Symptoms; SAS, Simpson–Angus Rating Scale of EPS; TAU, treatment as usual; TDO, tryptophan 2,3-dioxygenase; TNFα, tumor necrosis factor alpha
