# Supplementary material for: Neurobiology and Therapeutic Potential of Cyclooxygenase-2 (COX-2) Inhibitors for Inflammation in Neuropsychiatric Disorders
Source: Front Psychiatry. 2019 Sep 4;10:605. doi: 10.3389/fpsyt.2019.00605 (PMC6738329; doi:10.3389/fpsyt.2019.00605)
Supplement: Supplementary file 1 [file DataSheet_1.docx]

**Appendix A**

We used the following search terms to search all trials registers and databases:

Psychiatry OR neuropsychiatry OR depression OR major depressive disorder OR affective disorder OR bipolar disorder OR anxiety OR obsessive compulsive disorder OR autism OR autism spectrum disorder OR schizophrenia OR psychosis AND anti-inflammatory drugs OR non-steroidal anti-inflammatory OR cyclooxygenase-2 inhibitor OR cyclooxygenase 2 inhibitor OR prostaglandin synthase OR celecoxib OR rofecoxib OR parecoxib OR valdecoxib OR lumiracoxib OR etoricoxib OR firocoxib OR cimicoxib AND inflammation OR neurinflammation OR neurodegeneration OR neuroprotective.

**PRISMA 2009 Flow Diagram**

## **Screening**

## **Included**

## **Eligibility**

## **Identification**

Full-text articles assessed for eligibility
**(*N* = 28)**

Full-text articles excluded, with reasons
**(*N* = 8)**

Studies included in qualitative synthesis
**(*N* = 18)**

Studies included in quantitative synthesis/ meta-analysis
**(*Not applicable*)**

Records identified through database searching

**(*N* = 700)**

Additional records identified through other sources

**(*N* = 13)**

- Hand-picked (*n* = 3);
- Clinicaltrials.gov (*n* = 7);
- Stanley Med. Res. Inst. (*n* = 3).

Records after duplicates removed
***(N* = 698)**

Records screened
***(N* = 698)**

Records excluded
***(N* = 672)**
